# Influence of storms on the emission of pollutants from sewage into waters

**DOI:** 10.1038/s41598-021-97536-5

**Published:** 2021-09-22

**Authors:** Monika Suchowska-Kisielewicz, Ireneusz Nowogoński

**Affiliations:** Institute of Environmental Engineering, University of Zielona Gora, Licealna 9, 65-417 Zielona Góra, Poland

**Keywords:** Environmental impact, Environmental monitoring

## Abstract

During heavy precipitation, chemical and biological pollutants from urban and agricultural areas enter the waters from storm overflows as a result of infiltration and inflow, as well as via uncontrolled outflows from water treatment plants. Infiltration and inflow of rainwater into sewers is an especially popular and major worldwide problem. Climate forecasts indicate changes in climatic conditions towards an increase in the intensity and frequency of torrential rainfalls. It may therefore be assumed that the negative impact of rainwater on water quality will increase. This article attempts to address the question of the impact of pollution from wastewater introduced into water during rainy weather to the receiver. The assessment of the impact of rainfalls on a receiver was carried out on the basis of a simulation of pollution loads from sewage introduced into a river by storm overflows based on data from monitoring the amount of rainfall and simulating the operation of storm overflows using Environmental Protection Agency Storm Water Management Model (EPA SWMM). The obtained results were compared with the pollutant loads discharged at the same time from the sewage treatment plant (STP). In addition, the article assesses possible improvement solutions to reduce the negative impact of storm overflows on water.

## Introduction

Rational management of rainwater, especially in highly urbanized areas, can contribute in a significant way to water protection.

Precipitation is one of the sources of pollution influencing ecosystems through processes of eutrophication and acidisation of soil and waters^1,2^. This is related to the presence of substances such as sulfur dioxide, nitrogen oxides and ammonia in the air.

The results of studies on the chemistry of atmospheric precipitation and the deposition of pollutants into soil, conducted in Poland in the last 14 years, indicate a gradual decrease in sulphate deposition. However, no clear downward trend has been observed in the case of eutrophic pollutants (mainly nitrogen compounds).

The negative impact of precipitation on the condition of waters is dependent on the duration of precipitation, its intensity and the length of preceding rainless periods.

During heavy precipitation, chemical and biological pollutants from urban and agricultural areas enter the ground waters as a result of surface runoff—from storm overflows as a result of infiltration and inflow, as well as via uncontrolled outflows from water treatment plants^3^.

Infiltration and inflow of rainwater into sewers is an especially popular and major worldwide problem In Poland and Europe, most municipalities are served with combined sewer systems, which poses serious problems due to influx and infiltration^4^.

The size of infiltration and inflow varies widely in different countries, due to the complexity of sewage systems and climatic (rainfall intensity) and hydrogeological conditions, (Table 1). Climate forecasts indicate changes in climatic conditions towards an increase in the intensity and frequency of torrential rainfalls^5,6^. It may therefore be assumed that the negative impact of rainwater on water quality will increase^7,8^.

**Table 1:** Average estimated share of infiltration water and inflow in the sewage network^3^.

Country	%	References
Poland	47	Bugajski et al., 2016
Germany (Baden-Wurttemberg	32	Weiss et al., 2002
Netherlands	38	Schilperoort, 2004
Norway (14 different cities)	67	Odegaard, 2016
Austria (32 WWTPs)	25–50	Ertl et al., 2008
Sweden	50	Svensson et al., 1996
UK	45	White et al., 1997
Scotland (Edinburgh)	60	GDSDS, 2005
Ireland (Dublin) 10–75	10–75	GDSDS, 2005
Switzerland	35–65	Kracht et al., 2005
Canada	8	Holeton et al., 2011
USA	55–65	Pearlman, 2007

The amount of pollutants introduced into the receiver during rainfalls depends on the nature of the catchment area, the technical condition of the sewage system, the technical and technological solutions applied in the sewage treatment plant and the meteorological conditions of a given area.

Raw sewage diluted with rainwater and carrying suspended solids accumulated in the sewage system are introduced into the surface waters through storm overflows.

Discharges of sewage into ground waters occurring during the first flushing period, introducing a very high concentration of biological oxygen demand (BOD_5_) and toxic ammonia and hydrogen sulphide, are particularly burdensome to the receiver^9^.

An increase in the load of nitrogen in the ammonium form introduced to waters causes indirect N_2_O emissions to the atmosphere, enhancing the negative impact of precipitation on the environment^10^.

The amount of sewage discharge from storm overflows is limited by law. In Poland, it should not exceed 10 during the year. In reality, this amount is significantly greater. In Poland, in 2013–2019, the amount of discharge from overflow of storm sewer of the combined sewerage system of the left bank of Warsaw was 45 times higher than it is allowed by the legal water permit (the additional amount of sewage discharged into the Vistula without a permit amounted to over 5 million m^3^). The situation is similar in other cities (e.g. the Łódź network has 50 discharges per year, while 10 discharges per year are allowed)^11^. This phenomenon is present in 108 cities in Poland. The situation is similar in all of Europe.

The operation of storm overflows and storm water drainage has a large impact on the quality of surface waters^12–15^. Despite the modernization of old sewage treatment plants and construction of new ones in Poland, the condition of Polish rivers is still sub optimal. According to the report from 2018^16^, only 0.5% of rivers and dam reservoirs have achieved very good notes, and 16% a good ecological condition. Among artificial and heavily changed surface waters, the maximum potential was reached by 0.6%, and good—by 23%.

The threats resulting from the operation of storm overflows are caused both by the load of pollutants entering the receiver and its sanitary contamination. Wastewater, especially municipal sewage, discharged into the receiver, disturbs its ecological balance, e.g. as a result of an uncontrolled increase in the water volume flow, a decrease in the dissolved oxygen content and an increase in the amount of suspended solids and nitrogen and phosphorus compounds in the water. In addition, they introduce a large number of microorganisms, including pathogenic species, into the waters. The amount of pollutants and microorganisms in mixed municipal sewage and rainwater mainly depends on the course and dynamics of precipitation^17,18^. The duration of the dry spells, preceding the rainfall, during which sediment layers build up on the surface of canal bottom, may also be an important factor. Due to the increase in the share of sealed surfaces in cities, storm overflows are now very often activated even with relatively low intensity rains, causing a significant threat to the quality of the receiving waters^11^.

Growing problems related to ensuring good ecological status of waters lead to global application of an integrated approach, in which the impact of rainwater on the receiver is analyzed not only by analyzing storm overflows but also the impact of pollutant discharges from sewage treatment plants in this period^19,20^.

During heavy rainfall, the amount of sewage supplied to the treatment plant increases, which leads to exceeding the hydraulic volume of the treatment plant facilities, especially when these lack a storage reservoir. This adversely affects the effect of wastewater treatment. This is mainly related to the rinsing of the activated sludge from biological reactors, which may reduce the concentration of migroorganisms in the reactor (a parameter responsible for the effectiveness of wastewater treatment) and deterioration of the secondary settling tank operation, resulting in an increase in the concentration of suspended solids and other indicators of contamination in the treated wastewater discharged into a receiver^21^.

Moreover, dilution of sewage with water causes a significant decrease in sewage temperaturę (in the autumn and winter period), pH and BOD_5_ and a decrease in the ratio of BOD_5_/N and BOD_5_/P. Therefore, the nitrification process is deteriorated or completely inhibited^9,22^.

This article attempts to address the question of the impact of pollution from domestic and economic sewage introduced into waters during rainy weather on water quality. The assessment of the impact of heavy rainfalls on a receiver was carried out on the basis of a simulation of the size of pollution loads from domestic and economic wastewater introduced into a river by storm overflows and from sewage treatment plants based on data from monitoring the amount of rainfall and simulating the operation of storm overflows as well as the quantity and quality of treated wastewater, respectively. There is little information in the literature that compares the size of loads introduced to waters by storm overflows with those introduced to waters from sewage treatment plants. The article completes the knowledge in this field. Moreover, the article can be an interesting source of information for sewage network operators and sewage treatment plants and for designers, showing the practical use of the EPA model and pointing to problems and possible solutions in the operation of a combined sewage network.

## Research area

The analysis of the impact of precipitation on the emissions into the receiver of pollutants from domestic sewage was carried out for the village of Głogów, a medium-sized settlement inhabited by approximately 67,000 citizens. The sewage network in the catchment area under consideration serves Głogów and other small towns in the agglomeration. The number of people in the agglomeration is approximately 77,000.

### Sewerage network

The network discharging household sewage to the treatment plant consists of a mixed sewage system.

The older part of the collective sewage system is a combined system, and the newer part is a separate sewage system. The combined sewage system with a length of approx. 111 km is constructed of concrete, plastic and brick pipes, with circular, oval, elliptical, rectangular and numerous atypical sections.

The separating sewerage with a length of approx. 10.8 km has been constructed in modern times, mainly of plastic and GRP pipes.

**Figure 1:**
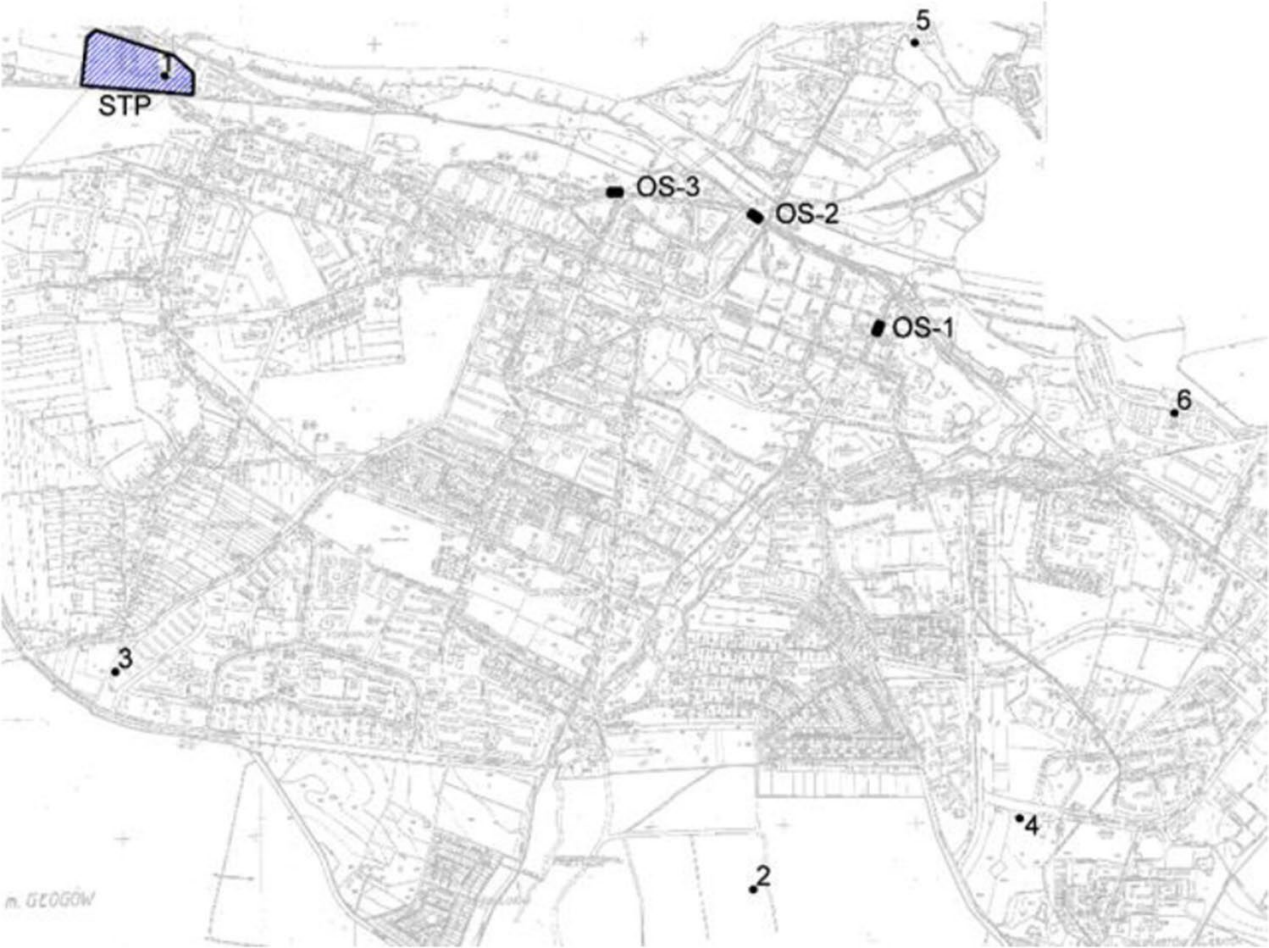
Arrangement of rain gauges and storm overflows on the sewage network in the area of the considered catchment. OS-1 ÷ 3—overflow structures; 1–6—rain gauges; STP—sewage treatment plant.

There are 3 typical storm overflows on the sewage system (OS-1, OS-2 and OS-3) (Fig. 1). The overflows are lateral, one-sided with a low overflow edge without choking elements. An additional storm overflow (RT) was located in the sewage treatment plant (STP). It is part of the inflow regulation system to the sewage treatment plant and, due to the fact that the excess sewage is directed to the retention reservoir, it is not subject to legal restrictions. The aforementioned retention reservoir has an emergency overflow that prevents overflowing of the facility. The outflow through the emergency overflow takes place directly to the receiver, through an outlet shared with the outflow of sewage treated by the sewage treatment plant.

There are 6 rain gauge stations located in the discussed area (Fig. 1).

### Sewage treatment plant

The sewage treatment plant in Głogów is a mechanical and biological treatment plant, in which organic and biogenic compounds are removed by means of activated sludge and sewage sludge treatment processes are carried out. The receiver of sewage is the Oder river, at km 395 + 275. The sewage treatment plant is supplied with domestic and industrial wastewater, with parts of rainwater or snowmelt from the city.

The maximum hydraulic capacity of the treatment plant is 1500 m^3^/h. In daily terms, the average capacity is 15,000 m^3^/d, and the maximum is 27,000 m^3^/d. Currently, the wastewater treatment plant in dry weather is loaded to the capacity of about 75%.

## Research methodology

### Determination of pollutant loads

Pollutant loads dumped into the receiver during the rainy season were designated separately for storm overflows and for sewage treatment plants.

Pollutant loads from storm overflows were determined with the use of a model applying the EPA SWMM program. The model was calibrated on the basis of a measurement campaign carried out in 2011–2013 in the scope of: the amount of rainfall measured in 6 rain gauge stations located in the catchment area and the sewage treatment plant (Fig. 1), parameters characterizing the work of overflows, the amount of sewage in the sewage system and in the inflow to the sewage treatment plant and the quality of sewage in the inflow to the sewage treatment plant determined in dry weather.

Loads introduced into the receiver from the treatment plant were determined on the basis of measurements of the amount of sewage and the results of qualitative tests of treated sewage in 2012–2014. These loads were calculated on the basis of the average monthly concentrations of total suspended solids (TSS), chemical oxygen demand (COD), biological oxygen demand (BOD_5_), total nitrogen (TN) and total total phosphorus (TP) as well as the daily amounts of sewage from the monitoring carried out at the sewage treatment plant.

### Modeling of the quantity and quality of wastewater introduced to the receiver by storm overflows

As a modelling tool for the described studies, the EPA (SWMM) model of managing stormwater was chosen. SWMM was developed, above all, for urban areas, enabling the simulation of short- and long-term quantity and quality of water^23^. Conceptually speaking, catchments in the SWMM are treated as non-linear tanks, which receive inflows from rainfalls and bordering catchments, generating various outflow components and losses, such as: surface runoff, infiltration and evaporation. The surface retention represented by: ponding, surface wetting and interception described the capacity of these tanks^24^. The runoff is calculated using Manning’s equation. Based on the obtained data, a model was developed based on^25^:

- Nodes—499 junctions and 4 outlets;
- Conduits—504 channel segments;
- Rainfall data—6;
- Rainfall catchments—370 catchments;
- Number of overflow structures—4.

The model was calibrated based on the results of field studies carried out from 1 September 2012 to the end of October 2014, using standard calibration parameters. The selection of key calibration parameters was based on the sensitivity analysis of the model^26^. The hydraulic catchment width was chosen as the basic calibration parameter and the mean catchment slope as a supplementary parameter.

The measurements covered the runoff depth within the overflow crests in three storm overflow structures, as well as the depth and intensity of flows in the inlet and outlet overflow channels. Six rain measurement stations were located within the area of the city.

Filling measurement in the overflow chambers was performed using ultrasonic devices. The probes were positioned over the overflow crests so they acted as a signaling device for the overflow occurrence and realized the measurement of the runoff depth on the overflow crest. Dry weather data was recorded every 1 min, and in rainy periods—every 15 s.

Measurements of filling and flow rate were carried out using an ADFM flow meter, dedicated for measuring and recording the flow of wastewater in partially and fully filled collectors^27^. Measurements were performed using the "Range Gated" hydroacoustic measurement technique cooperating with a 5-beam measuring probe, which made it possible to determine the actual speed profile in the measurement cross-section. The speed measurement was carried out by means of 2 pairs of sensors, projecting beams in 4 different oriented directions at an angle of 10˚ and 20˚. Depth was determined on the basis of the propagation time of a 5th, vertically projected beam. The flow rate, along with the speed and depth, was continuously recorded in the memory of the device. The recovery time of the reflected signal coming from different distances from the probe was calculated using the known speed of propagation of the ultrasonic wave in the medium. This solution made it possible to determine the average velocity for individual measuring cells.

ADFM flow meters were installed:

- Overflow OS-1—on the inlet sewer and outflow sewer (continuation flow) from the overflow;
- Overflow OS-2—on the two inlet sewers and outflow sewer (continuation flow) from the overflow;
- Overflow OS-3—on the outflow sewer from the overflow (continuation flow) and the spill flow sewer;
- At the sewage treatment plant (STP)—in the emergency overflow sewer of the retention tank;
- In the relief canal connecting the inflow sewer to the OS-1 facility and one of the OS-3 inflows.

Data were recorded every 2 min, except for the flow meter at the wastewater treatment plant (in which case data were recorded every 5 min).

Dry weather data was recorded every 1 min, and in rainy periods—every 15 s. Digital communication between the sensor and the measuring module ensured immunity to electromagnetic interference. A minimal "dead zone" in the filling measurement kept the readings stable.

The flow meter also recorded diagnostic parameters, i.e. signal strength and percentage of correct results, which enables easy control of the probe's operation and optimization of the location of the measurement point.

The rain gauge measurement was carried out using automatic weighing rain gauges with data recorders with a sensitivity of 0.1 mm and measurement accuracy of 0.1% (data was recorded every 1 min). The measurement data was transmitted wirelessly via the mobile telephone network to the operator's console in the sewage treatment plant^27^.

The proper functioning of the model described above was verified using the results of studies carried out in Głogów by the Institute of Environmental Engineering (University of Zielona Góra) in the years 1998–2000. Archival research was carried out at the stage of modernizing the sewage treatment plant in Głogów^28^. It served, among others, to verify the capacity of the retention tank located directly before the treatment plant, enabling sewage containing outflows from the first run-off wave from the surface of the catchment to be contained.

The studies were realized by the measurement of the runoff levels (depths) in channels in six selected gauging sections.

The verification of the simulation results based on field measurements was assessed based on the relative residual error RRE estimated as^29^:

1 $$RRE=\frac{\sqrt{\sum_{i=1}^{n}{\left({Q}_{p,i}-{Q}_{o,i}\right)}^{2}}}{\sum_{i=1}^{n}{Q}_{o,i}}$$

where: Q_o_—calculated flow rate; Q_p_—measured flow rate.

Model validation against archival data showed satisfactory results in more than 60% of cases. Especially since the archival rain gauge data did not allow for spatio-temporal variability of precipitation^14^.

Model validation against archival data showed satisfactory results in more than 60% of cases. Especially since the archival rain gauge data did not allow for spatio-temporal variability of precipitation^14^.

Model verification based on selected 2014 precipitation also confirmed the applicability of the model. Performance analysis was based on 11 phenomena (recorded data at from 1 to 5 measurement sites). A total of 31 data series were analyzed and confronted with simulation results. Evaluation was performed based on relative residual error (RRE)^25^, Pearson correlation coefficient (R) and Nash efficiency coefficient (E) (Table 2). In the case of relative residual error, a very good result^29^ was obtained in 2 cases, with a good result also noted in 11; in 10 cases the result was assessed as average, whereas in 8 – unsatisfactory. In the case of Pearson correlation coefficient, a good result was obtained in 7 cases; in 10 cases the result was assessed as average, whereas in 5 – unsatisfactory. 9 results were unacceptable. In the case of Nash efficiency coefficient, an excellent^30,31^ result was obtained in 2 cases, with a good result also noted in 5; in 8 cases the result was assessed as satisfactory, whereas in 4 – passable. 12 results were assessed as poor. Extreme variances was associated with the conduct of renovation work associated with local wastewater diversion. Moreover, the elevation of the overflow edges was corrected.

**Table 2:** Calibration verification model based on the following coefficients: relative residual error (RRE)^25^, Pearson correlation coefficient (R), Nash efficiency coefficient (E).

Date of occurrence	Verification coefficient	Skargi street	Nadbrzeżna street			Towarowa street	
		Measurement stations					
		1.3	2.1	2.2	2.3	3.1	3.2
24.05.2014	RRE	7.8	−	−	5.4	−	−
	R	0.91	−	−	0.9	−	−
	E	0.74	−	−	0.36	−	−
27.05.2014	RRE	−	−	−	19.8	−	−
	R	−	−	−	0.62	−	−
	E	−	−	−	−2.39	−	−
28.05.2014	RRE	6.8	7.1	−	1.3	1.7	−
	R	0.98	0.95	−	0.98	0.88	−
	E	0.05	−2.24	−	0.79	0.17	−
24.06.2014	RRE	6.3	15.8	4.2	3.2	4.4	−
	R	0.91	0.33	0.95	0.88	0.86	−
	E	0.76	−7.58	0.89	0.77	0.64	−
09.07.2014	RRE	9.6	−	5.8	3.1	6.9	12
	R	0.92	−	0.95	0.76	0.73	0.14
	E	0.98	−	0.97	0.5	0.2	−26.8
03.08.2014	RRE	−	−	−	5.1	−	−
	R	−	−	−	0.86	−	−
	E	−	−	−	0.02	−	−
05.08.2014	RRE	−	−	6.1	−	−	−
	R	−	−	0.91	−	−	−
	E	−	−	0.62		−	−
05.08.2014	RRE	−	−	5.3	−	−	−
	R	−	−	0.92	−	−	−
	E	−	−	0.62	−	−	−
09.09.2014	RRE	−	−	9.1	9.0	−	20
	R	−	−	0.95	0.92	−	0.39
	E	−	−	0.86	0.81	−	−3.35
11.09.2014	RRE	10	11.9	4	3.8	13.5	−
	R	0.91	0.36	0.91	0.95	0.62	−
	E	0.43	−1.65	0.78	0.88	−4.05	−
20.09.2014	RRE	−	19.5	−	4.8	20	−
	R	−	0.36	−	0.92	0.88	−
	E	−	−5.34	−	0.92	0.44	

### Main computational principles of the hydraulic model

The simulation model applied was based on the Dynamic Wave method, which, compared to Steady Flow and Kinematic Wave methods, is numerically more complicated, but most similar to real conditions and usually used in this type of realizations^26^. The method is based on the basic relationships known as the St. Venant equations^23^:

2 $$\frac{\partial A}{\partial t}+\frac{\partial Q}{\partial x}=0$$

3 $$\frac{\partial Q}{\partial t}+\frac{\partial \left({Q}^{2}/A\right)]}{\partial x}+gA\frac{\partial H}{\partial x}+gA{S}_{f}=0$$

where x—distance; t—time; A—flow cross-sectional area; Q—flow rate; H—hydraulic head of water in the conduit; Z—conduit invert elevation; Y—conduit water depth; S.f—friction slope (head loss per unit length); g—acceleration of gravity.

The derivation of these equations can be found in standard texts. The assumptions on which they are based are:

- Flow is one dimensional.
- Pressure is hydrostatic.
- The cosine of the channel bed slope angle is close to unity.
- Boundary friction can be represented in the same manner as for steady flow.

The continuity equation can be combined with the momentum equation to produce the following form of the momentum equation for a conduit^23^:

4 $$\frac{\partial Q}{\partial t}=2U\frac{\partial A}{\partial t}+{U}^{2}\frac{\partial A}{\partial x}-gA\frac{\partial H}{\partial x}-gA{S}_{f}$$

While this equation can be used to compute the time trajectory of flow in a conduit, another relationship is needed to do likewise for heads. Two types of nodes are possible. Non-storage junction nodes are assumed to be points with zero volume and surface area while storage nodes (such as ponds and tanks) contain both volume and surface area.

Each “node assembly” consists of the node itself and half the length of each link connected to it. Conservation of flow for the assembly requires that the change in volume with respect to time equal the difference between inflow and outflow. In equation terms^23^:

5 $$\frac{\partial V}{\partial t}=\frac{\partial V}{\partial H}\frac{\partial H}{\partial t}={A}_{s}\frac{\partial H}{\partial t}=\sum Q$$

where V—node assembly volume; A_s_—node assembly surface area; ΣQ—net flow into the node assembly (inflow-outflow).

Equations provide a coupled set of partial differential equations that solve for flow Q in the conduits and head H at the nodes of the conveyance network. Because they cannot be solved analytically a numerical solution procedure must be used instead.

### The main computational principles of the water quality model

The qualitative analysis was based on the inflow of sanitary sewage, excluding the accumulation and washing of pollutants from the catchment area. The model implements a one-dimensional model of pollution transport along the canal, described by the equation^23^:

6 $$\frac{\partial c}{\partial t}=-\frac{\partial \left(uc\right)}{\partial x}+\frac{\partial }{\partial x}\left(D\frac{\partial c}{\partial x}\right)+r\left(c\right)$$

where c—constituent concentration; u—longitudinal velocity; D—longitudinal dispersion coefficient; r (c)—reaction rate term; x—longitudinal distance; t—time.

The solution process is very difficult because there is one such equation for each pipe and channel in the conveyance network. These are linked together by the boundary conditions. The result is a large system of algebraic differential equations that must be solved simultaneously.

### Receiver specifications

Sewage from storm overflows and treatment plants is discharged to the Oder River.

The maximum flow with a probability of 0.2%, 1% and 10%, as well as the average flow on the Oder section at the level of Głogów, according to the data of the Institute of Meteorology and Water Management for the multi-year water gauge from 1951–2020, are as follows:

- SSQ: 198 m^3^/s.
- SNQ: 73,9 m^3^/s.
- SWQ: 731 m^3^/s.
- Qmax,0,2%: 2875 m^3^/s.
- Qmax,1%: 2078 m^3^/s.
- Qmax,10%: 1185 m^3^/s.

The current chemical composition of the Oder at the site of sewage discharge from overflows and from treatment plants (surface water bodies (JCWP): Oder from the Eastern Channel to the Czarna Struga, Rzuchowska Struga, Dalkówka, Biegnica, Głogowski channel and Rudna from Moskorzynka to the Oder), was assessed as strongly altered and in bad condition, at risk of not achieving the environmental objective of good ecological potential and good chemical condition.

In accordance with the water permit, pollutants of the following sizes may be introduced into the Oder River along with treated sewage:

- COD: 1875 kg/d.
- BOD_5_: 225 kg/d.
- TSS: 225 kg /d.
- TN: 150 kg/d.
- TP: 15 kg/d.

## Influence of rainwater on the load of pollutants introduced into the receiver

### The amount of sewage introduced into the receiver

**Figure 2:**
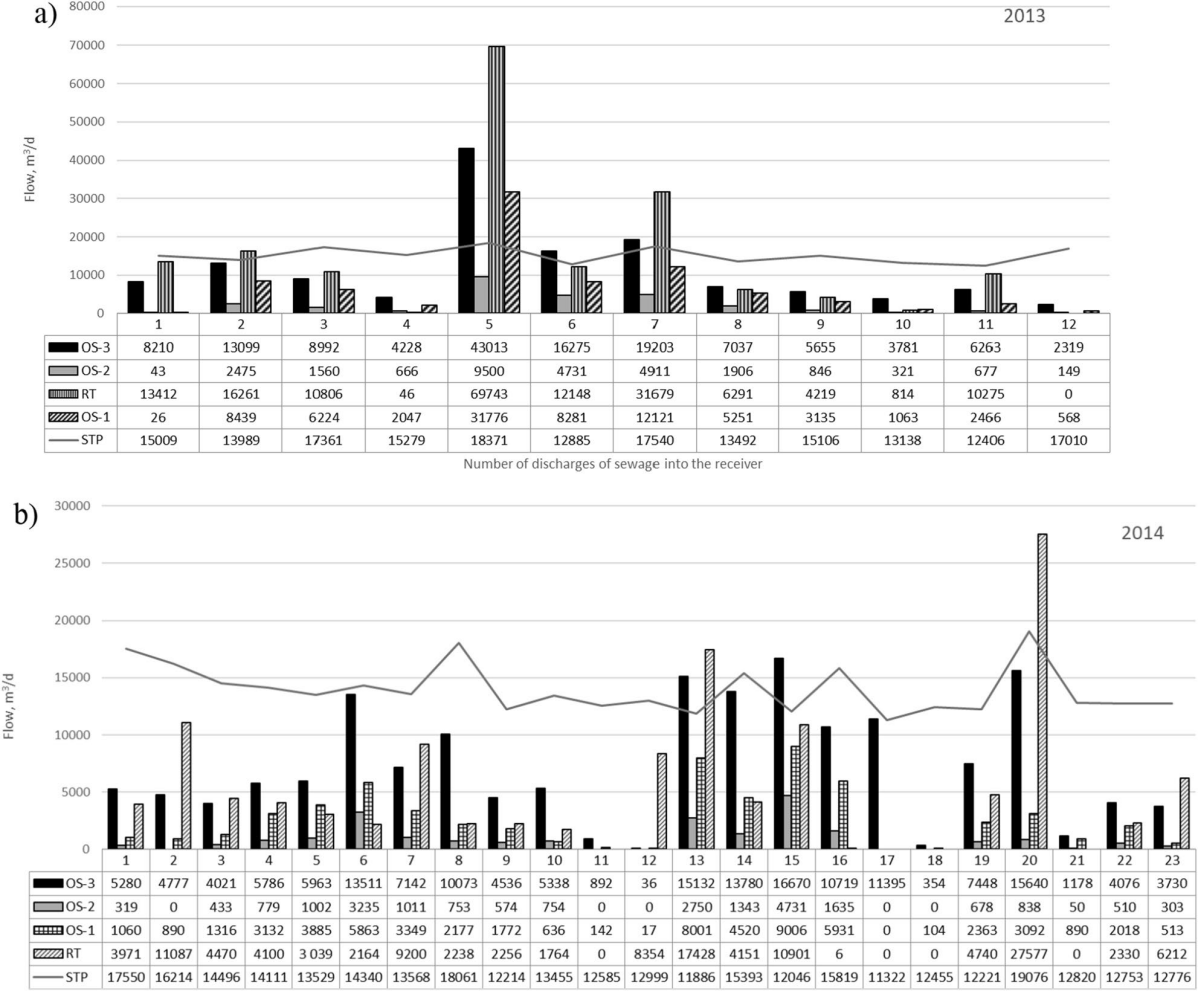
The amount of sewage discharged into the Oder River from overflows and from the treatment plant in (**a**) 2013 and (**b**) 2014.

In 2013 and 2014, 12 and 25 intensive rainfall, respectively, were recorded, as a result of which untreated sewage diluted with rainwater was discharged into the receiving body. Simulations of the amount of sewage introduced into the river from individual overflows are shown in Fig. 2. The gray line on the figure also displays the amount of sewage introduced to the receiver together with treated sewage, determined on the basis of actual measurements.

**Figure 3:**
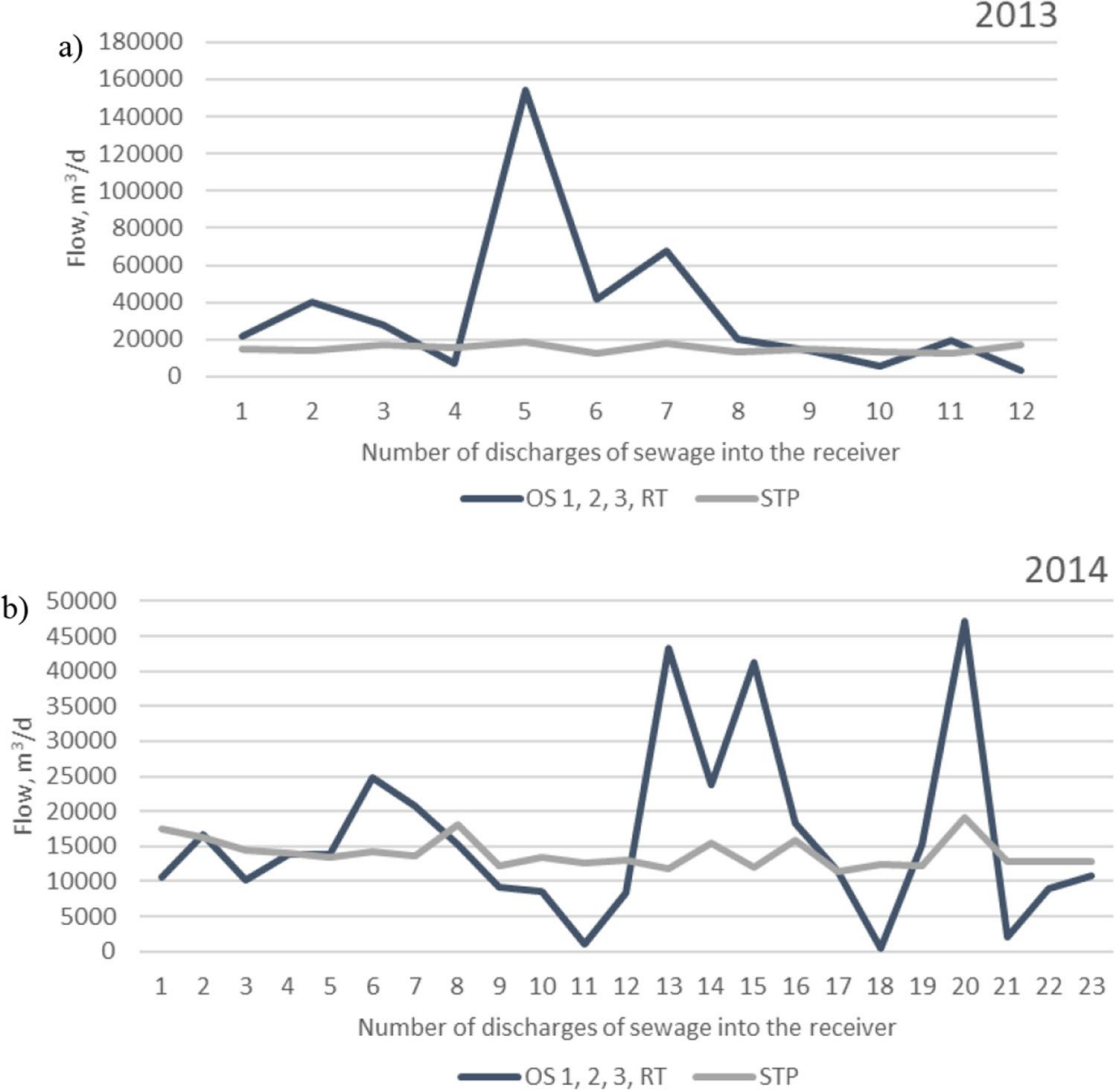
The total amount of discharge to the receiver of sewage from overflows and the amount of sewage discharged from the treatment plant in the years (**a**) 2013 and (**b**) 2014.

Figure 3 shows the total amount of sewage discharge from the overflows OS 1–3, RT and the amount of treated sewage discharged from the treatment plant at the same time in the years a) 2013 and b) 2014.

Analysis of the amount of sewage discharged from storm overflows to the Oder demonstrates that the amount of sewage introduced from individual overflows was periodically significantly higher than the amount of sewage introduced by the treatment plant (Fig. 2).

Considering the total amount of sewage introduced from all overflows to the receiver, it was even 7 times higher in 2013 and 3 times higher in 2014 (Fig. 3) from the amount of sewage introduced from the treatment plant within that period.

The presented results show that for the analyzed period the amount of sewage discharges by all overflows is higher than the permissible value, which in Poland for agglomerations < 100,000 PE is 10 times a year.

However, taking into account the second criterion used in Poland for agglomerations < 100,000 PE, related to the assessment of the overflow operation correctness based on the permissible flow (at least 400% of the average dry weather flow), it indicates that the overflows are functioning properly. The OS-1 overflow discharges 900% of the average runoff in dry weather towards the treatment plant, the OS-2 overflow discharges 1400% of the average runoff in dry weather, and the OS-3 overflow drains 410% of the average runoff in dry weather.

### Size of pollutant loads introduced into the receiver

The amount of pollutant loads introduced into the Oder from storm overflows OS1, OS2 and OS3 located on the sewage network and at the sewage treatment plant (RT), estimated on the basis of the model, are presented in Table 3.

**Table 3:** Pollution loads introduced into the Oder by storm overflows in 2013–2014 estimated on the basis of the model.

LP	2013	OS1	OS2	OS3	RT	OS1	OS2	OS3	RT	OS1	OS2	OS3	RT	OS1	OS2	OS3	RT
		TSS, kg / d				COD, kg / d				TN, kg / d				TP, kg / d			
1	28.05.13	1.12	1.17	303.00	225.87	2.97	3.08	800.55	596.77	0.18	0.18	47.84	35.66	0.04	0.04	9.66	7.13
2	30.05.13	173.52	24.77	282.53	305.58	458.46	65.45	746.49	807.36	27.40	3.91	44.61	48.25	5.48	0.782	8.92	9.65
3	03.06.13	85.04	12.15	169.53	132.16	224.68	32.10	447.43	349.18	13.43	1.92	26.74	20.87	2.69	0.348	5.44	4.17
4	04.06.13	62.06	10.47	147.00	560.00	163.97	27.66	388.39	1.47	9.80	1.65	23.21	0.09	1.96	0.331	4.64	0.02
5	25.06.13	373.40	63.01	496.54	586.90	986.57	166.48	1311.91	1550.66	58.96	9.95	78.40	92.67	11.79	1.990	15.68	18.53
6	29.07.13	28.46	12.43	93.30	119.37	75.20	32.84	246.50	315.39	4.49	1.96	14.73	18.85	0.90	0.392	2.95	3.77
7	30.07.13	60.23	16.04	122.76	171.26	159.13	42.39	324.34	452.49	9.51	2.53	19.38	27.04	1.90	0.507	3.88	5.41
8	07.08.13	68.50	9.36	87.19	71.47	180.98	24.73	230.36	188.82	10.82	1.48	13.77	11.28	2.16	0.296	2.75	2.26
9	09.08.13	73.30	13.43	157.05	60.84	193.67	35.49	414.93	160.75	11.57	2.12	24.80	9.61	2.32	0.424	3.88	1.92
10	13.09.13	39.68	8.06	136.12	11.99	104.83	21.28	359.64	31.68	6.27	1.27	21.49	1.89	1.25	0.254	4.30	0.38
11	14.09.13	41.38	7.56	139.81	108.74	109.32	19.97	369.38	287.31	6.53	1.19	22.07	17.17	1.31	0.239	4.42	3.43
12	17.09.13	14.65	2.77	59.48	0.00	38.70	7.32	157.17	0.00	2.31	0.44	9.39	0.00	0.46	0.087	1.88	0.00
**2014**																	
1	23.03.14	34.41	7.06	238.95	81.93	90.90	18.65	631.32	216.46	5.43	1.11	37.73	12.94	1.09	0.22	7.55	2.59
2	24.03.14	4.03	0.00	211.40	151.93	10.65	0.00	558.55	401.43	0.64	0.00	33.38	23.99	0.13	0.00	6.68	4.80
3	25.03.14	0.00	0.00	0.00	0.00	0.00	0.00	0.00	0.00	0.00	0.00	0.00	0.00	0.00	0.00	0.00	0.00
4	09.04.14	37.90	7.70	129.21	92.96	100.12	20.35	341.38	245.61	5.98	1.22	20.40	14.68	1.20	0.24	4.08	2.94
5	17.05.14	56.60	7.87	150.59	48.63	149.54	20.77	397.87	128.50	8.94	1.24	23.78	7.68	1.79	0.25	4.76	1.54
6	18.0514	63.70	10.09	114.14	34.91	168.30	26.67	301.56	92.23	10.06	1.59	18.02	5.51	2.01	0.32	3.60	1.10
7	24.05.14	60.74	13.25	124.30	19.17	160.49	35.01	328.42	50.64	9.59	2.09	19.63	3.03	1.92	0.42	3.93	0.61
8	27.05.14	80.69	16.32	207.90	132.82	213.18	43.12	549.28	350.93	12.74	2.58	32.83	20.97	2.55	0.52	6.57	4.19
9	28.05.14	54.72	12.67	262.01	263.13	144.57	33.47	692.25	695.22	8.64	2.00	41.37	41.55	1.73	0.40	8.27	8.31
10	24.06.14	48.17	9.64	117.47	32.45	127.28	25.46	310.38	85.74	7.61	1.52	18.55	5.12	1.52	0.30	3.71	1.03
11	25.06.14	44.80	16.81	138.41	34.05	118.38	44.40	365.69	89.97	7.07	2.65	21.85	5.38	1.42	0.53	4.37	1.08
12	30.06.14	7.25	0.00	56.11	0.00	19.17	0.00	148.24	0.00	1.15	0.00	8.86	0.00	0.23	0.00	1.77	0.00
13	08.07.14	0.99	0.00	4.07	0.00	2.64	0.00	10.74	0.00	0.16	0.00	0.74	0.00	0.03	0.00	0.13	0.00
14	09.07.14	74.27	15.77	246.40	124.83	196.22	41.68	651.03	329.80	11.73	2.49	38.91	19.71	2.35	0.50	7.78	3.94
15	24.07.14	91.35	16.53	374.18	241.05	241.32	43.66	988.61	636.88	14.42	2.61	59.08	38.06	2.88	0.52	11.82	7.61
16	03.08.14	50.52	16.11	154.31	34.02	133.47	42.57	407.70	89.88	7.98	2.54	24.37	5.37	1.60	0.51	4.87	1.07
17	05.08.14	95.82	15.16	213.33	163.05	253.17	40.05	563.63	430.80	15.13	2.39	33.68	25.75	3.03	0.48	6.74	5.15
18	06.08.14	0.00	0.00	0.00	0.10	0.00	0.00	0.00	0.27	0.00	0.00	0.00	0.02	0.00	0.00	0.00	0.00
19	07.08.14	0.00	0.00	44.67	0.00	0.00	0.00	118.02	0.00	0.00	0.00	7.05	0.00	0.00	0.00	1.41	0.00
20	31.08.14	5.25	0.00	26.18	0.00	13.88	0.00	69.18	0.00	0.83	0.00	4.13	0.00	0.17	0.00	0.83	0.00
21	01.09.14	67.28	12.96	226.06	51.78	177.77	34.24	597.27	136.80	10.62	2.05	35.69	8.18	2.13	0.41	7.14	1.64
22	02.09.14	84.45	14.92	421.11	345.90	223.12	39.42	1112.63	913.91	13.33	2.36	66.49	54.62	2.67	0.47	13.30	10.92
23	09.09.14	4.94	2.86	52.09	0.00	13.06	6.83	137.63	0.00	0.78	0.41	8.23	0.00	0.16	0.08	1.65	0.00
24	11.09.14	46.53	7.77	116.21	34.39	122.94	20.52	307.05	90.87	7.35	1.23	18.35	5.43	1.47	0.25	3.67	1.09
25	20.09.14	12.32	5.42	143.31	104.96	32.56	14.33	378.64	277.32	1.95	0.86	22.63	16.57	0.39	0.17	4.53	3.32

**Figure 4:**
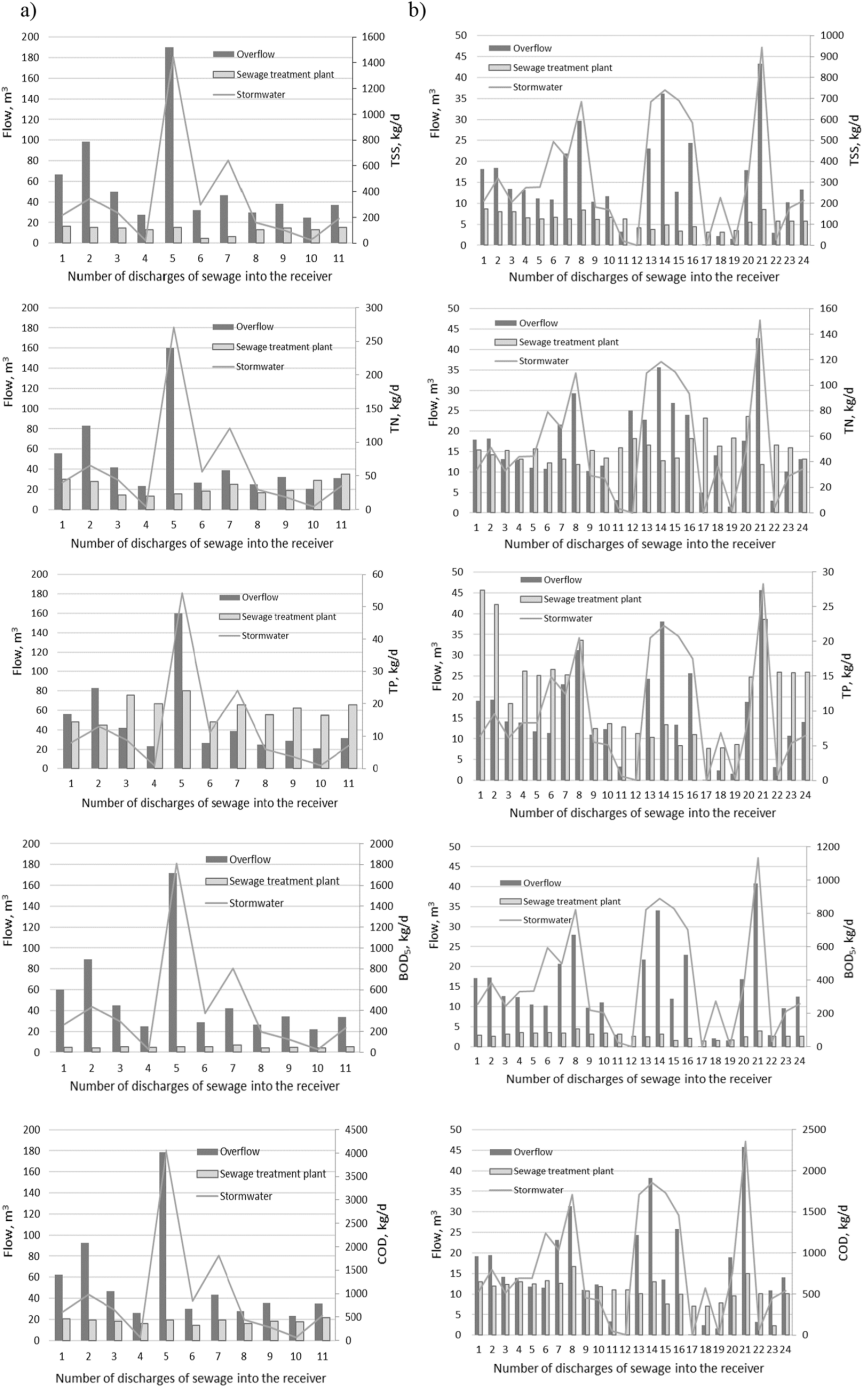
Total pollutants discharged into the Oder from storm overflows and treatment plants in (**a**) 2013 and (**b**) 2014.

Figure 4 shows the size of the total pollutants introduced into the Oder from the overflows of OS1, OS2, OS3 and RT in 2013 and 2014, and the total loads introduced with treated sewage during heavy rains. The grey line indicates the amount of sewage introduced to the receiver from overflows.

The data presented above demonstrates that, as a result of sewage discharges from overflows to the receiving body, pollutant loads were periodically many times higher than the concentrations of pollutants introduced (Table 3) together with treated sewage (Fig. 4) and the permissible loads that can be introduced by sewage treatment plants.

The highest exceedances of the permissible loads of pollutants introduced to the Oder in 2013 were recorded in the discharge of BOD_5_ and COD loads, which on June 25 (5th discharge of sewage from overflows) were respectively about 8 and 5 times higher, while the loads of TSS about 4 and TN and TP were about 2 times higher. In 2014, the highest exceedances of the permissible BOD_5_, COD and TSS loads were recorded on September 1 (24th discharge of sewage from overflow). They were, respectively, about 5 and 2 times higher than the permissible values (Fig. 3).

## Discussion

The condition of waters in Poland and other European countries is still suboptimal, hence reduction of pollutant loads discharged into waters during precipitation is very important^32^.

Regardless of the stormwater overflow design solution its impact is always adverse to the environment.

The discharge of sewage into the river contributes to processes that often lead to a reduction in dissolved oxygen or local anoxia^33^. Usually, the trophic state of reservoirs changes locally under the influence of inflowing sewage, which leads to a change in the water biocenosis^34^.

Factor that makes difficult to control the correct operation of storm overflows in Poland are unclear regulations. In the case of Głogów, which as an agglomeration serves less than 100,000 of real inhabitants, at present, a simplified method is used to assess the correctness of the operation of storm overflows, which is to ensure the outflow towards the treatment plant not less than 400% of the average dry weather runoff. However, taking into account the official planning documents, the work of storm overflows in Głogów should be assessed in accordance with the criteria used for agglomerations > 100,000 PE. In such a case, the condition of frequency of transfers should be met, which should not exceed 10 per year. This frequency should be confirmed with a simulation model. Therefore, the long-term task should not be to meet the current legal requirements and properly document them, but to reduce both the frequency and the size of the stream flowing to the receiver to a lower level technically and economically justified. The basis for these activities should be a systemic approach to control the operation of combined sewer systems based on continuous monitoring of rainfall and existing storm overflows, as well as predictions of network behavior based on modelling taking into account climate change^35^. And it is not only about the current control of, for example, the position of the overflow edge, but rather the control of the entire catchment and its equipment, the implementation of corrective actions, related to the construction of new facilities and the regulation of their parameters^36^.

Both the technical condition and technological and organizational solutions of the network and sewage treatment plants have a significant impact on the amount of loads discharged into the waters during rains. Due to the fact that there is usually no information on the physical—chemical composition of wastewater discharged from overflows to the receiver, the use of model data is necessary in the proper management and operation of a network and protection of the receiver.

Data presented in the article demonstrates the results of a simulation of the pollutant load volume discharged into the Oder, performed on the basis of the EPA SWMM model calibrated for the sewage network of the city of Głogów, Poland. The aim of the simulation was to assess the scale of environmental hazards occurring during heavy rains. Due to the fact that the sewage treatment plant is an integral part of the sewage system, the pollutant loads introduced by sewage treatment plants along with the sewage treated based on actual operational data were additionally taken into account.

The data presented in the article displays that during the period of torrential rains, organic and biogenic pollutants were introduced into the Oder that were significantly higher than those from sewage treatment plants (Fig. 4).

**Figure 5:**
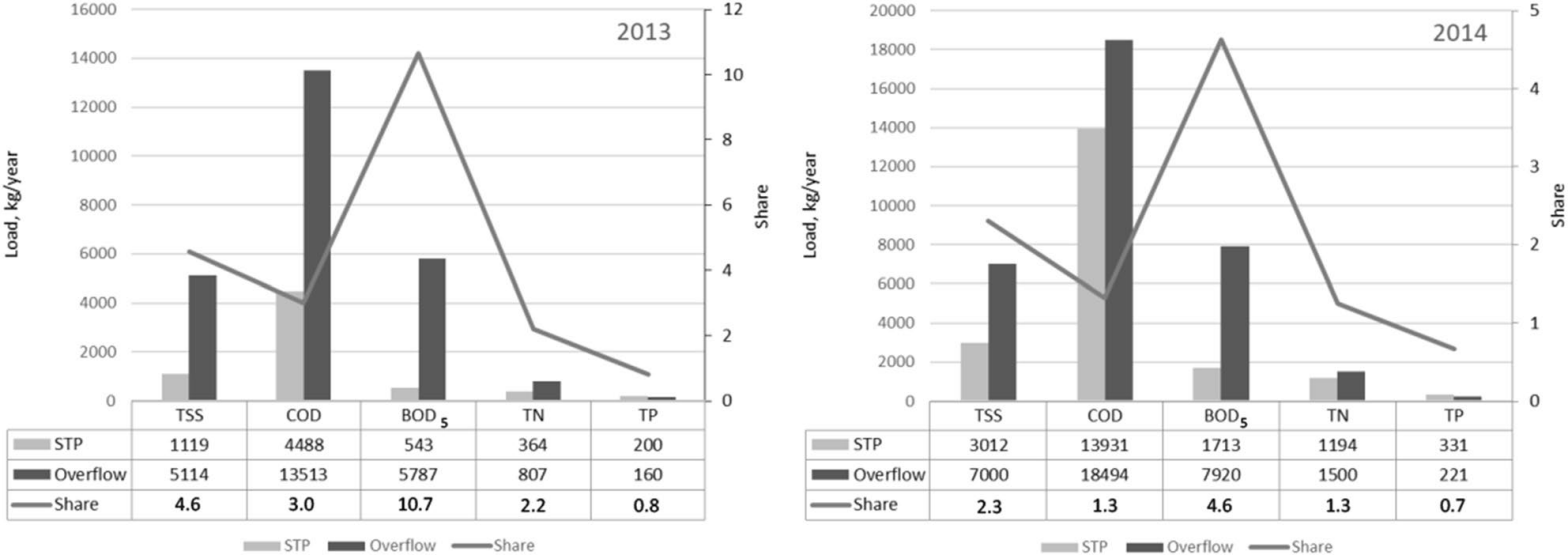
Annual loads of pollutants discharged into the Odra River during heavy rains in 2013 and 2014 from storm overflows and treatment plants.

Data analysis shows that the highest pollutant loads introduced into the Oder were of organic origin: BOD_5_ and COD, responsible for the oxygen conditions in the receiver. Biogenic pollutants (nitrogen and phosphorus) responsible for eutrophication were characterized by much lower values (Fig. 5).

Annual BOD_5_ load introduced to the receiver by storm overflows in 2013 and 2014 was respectively 10 and 5 times higher than annual BOD_5_ discharge from the treatment plant (Fig. 5). The section of the Oder at the site of wastewater discharge is considered to contain heavily changed waters with a bad chemical and biological condition. The introduction of such high additional loads of organic pollutants into the receiver limits the possibilities of improving the chemical and biological condition of the Oder. Additionally, the annual load of organic pollutants discharged into the Oder from the sewage treatment plant in Głogów in 2012–2018 demonstrates an upward trend^37^. Counteracting discharges from storm overflows and modernization of the network as well as improvement of the efficiency of the treatment plant operation is necessary in order to obtain the appropriate ecological state of the river.

Biogenic pollutants introduced by overflows in 2013 and 2014 are characterized by much lower values. Their annual share in relation to the N pollutants discharged into the Oder from sewage treatment plants was 2.2 and 1.3 times higher, respectively. However, the phosphorus load was lower. In 2013 it was 80% and in 2014—70% of the load discharged to the Odra River from the sewage treatment plant, respectively (Fig. 5).

The problem of infiltration and sewer inflow is common and is faced by many cities around the world^20^. The main cause of this phenomenon is dynamic urbanization, associated with uncontrolled sealing and expansion of the urban catchment area served. At the same time, the existing systems were not modernized, or unconsidered solutions were introduced, for example in the form of relief sewers, whose impact on the operation of the sewerage system was negligible. As a result, the occurrence of combined sewer overflows may be more frequent, in the case of less intense precipitation. Therefore, in order to protect water and the natural environment, it is extremely important to have a systemic approach to control the operation of combined sewer systems based on continuous monitoring of rainfall and storm overflows, as well as predictions of network behavior based on modeling. Due to the lack of discharge control, rainwater has a higher and higher negative impact on the environment, especially taking into account the cumulative effect of pollution and the increasing tendency in the amount of torrential rains in recent years. In assessing the degree of threats in this area, the technical condition as well as technological and organizational solutions for the operation of the network and sewage treatment plants should also be taken into account.

The results of the analysis of the possibility of reducing hydraulic load of the network in Głogów by using retention reservoirs and retention by using the capacity of channels with high slopes, which has not yet been published, do not allow to indicate the final optimum solution. Analysis of other objects^36^ indicates the necessity of applying complex, multipath actions including: retention, infiltration, and sustainable storm water management targeted at the retention of rain in the place of its origin. In the last case, rainwater is not discharged into the sewer system and a much smaller its part thanks to the use of natural retention is discharged into retention reservoirs. As a result, not only is the frequency of overflows reduced, but also the volume of excess runoff when it occurs. A new direction of research in this area is the creation of hybrid concepts combining the traditional approach with sustainable methods of rainwater management in the urban system.

## Summary

The data presented in the article confirm that the proper management of stormwater discharged into the reservoir is of significant importance in shaping the quality of water and the natural environment. The volume of pollutants discharged from storm overflows to the receiver during the period of heavy rains was many times greater than the loads discharged to the receiver from the treatment plant. It should be emphasized that, in addition to organic and biogenic pollutants, also sanitary pollutants, including pathogenic microorganisms, are discharged from overflows into the waters.

Due to climate change, the negative impact of storm overflows on the receiver will increase.

The main problem under climate change is the change in the specificity of precipitation both in Europe^38^ and western Asia^39^. Extreme rainfall is forecasted with a simultaneous increase in rainless periods. A single outflow from an overflow will potentially have a larger volume, while a longer rainless period and higher intensity of precipitation will result in additional load of pollutants flushed from the catchment surface^12^ and washed from the sediments accumulated in the canal network.

An optimum solution would be to separate the combined sewer system into independent sanitary and stormwater sewerage. Due to the fact that the majority of the combined sewer system in Poland serves the oldest, often historic, areas of the city and densely built-up areas, this solution is expensive and difficult to implement.

Another solution is to reduce inflow to storm overflows by delaying inflow through retention basins and unused retention capacity in the sewer network. An alternative solution is to reduce stormwater inflow to the sewer system by infiltration in infiltration basins, collection for later use and other techniques such as Low Impact Development (LID)^42^ or Sustainable urban drainage systems (SUDS)^42^. The analyses performed so far indicate that the synchronous use of LID techniques with classical solutions such as retention in tanks or retention in sewers^41–45^ is optimal.

The using of sewer network operation modeling is necessary to determine the changes in the magnitude of pollutant loads that can be discharged to the receiver from the sewer network after the implementation of remedial actions. This is important in order to counteract the negative effects of wastewater discharges into receiving waters from overflows because wrong decisions may displace the problem instead of reducing it.

## Data Availability

The datasets used and analysed during the current study are available from the corresponding author on reasonable request.
